# Elucidation of the Off-Center Displaced Mo in Octahedral Coordination in Ba_2_MoO_5_

**DOI:** 10.1021/acs.inorgchem.4c03617

**Published:** 2025-01-01

**Authors:** Andries van Hattem, Laurent de Geus, Ana Sacristán, Robert Dankelman, Sebastian Couweleers, Christoph Hennig, Jean-Christophe Griveau, Eric Colineau, Kathy Dardenne, Jörg Rothe, Tim Pruessmann, Rudy J. M. Konings, Anna L. Smith

**Affiliations:** † Radiation Science & Technology Department, Faculty of Applied Sciences, Delft University of Technology, Mekelweg 15, Delft 2629JB, The Netherlands; ‡ ESRF, the European Synchrotron, 71 Avenue des Martyrs, Grenoble Cedex 9, CS40220, 38043, France; § European Commission, Joint Research Centre, Karlsruhe D-76125, Germany; ∥ Institute for Nuclear Waste Disposal (INE), Radionuclide Speciation Department, Karlsruhe Institute of Technology (KIT), Hermann-von-Helmholtz-Platz 1, Eggenstein-Leopoldshafen 76344, Germany

## Abstract

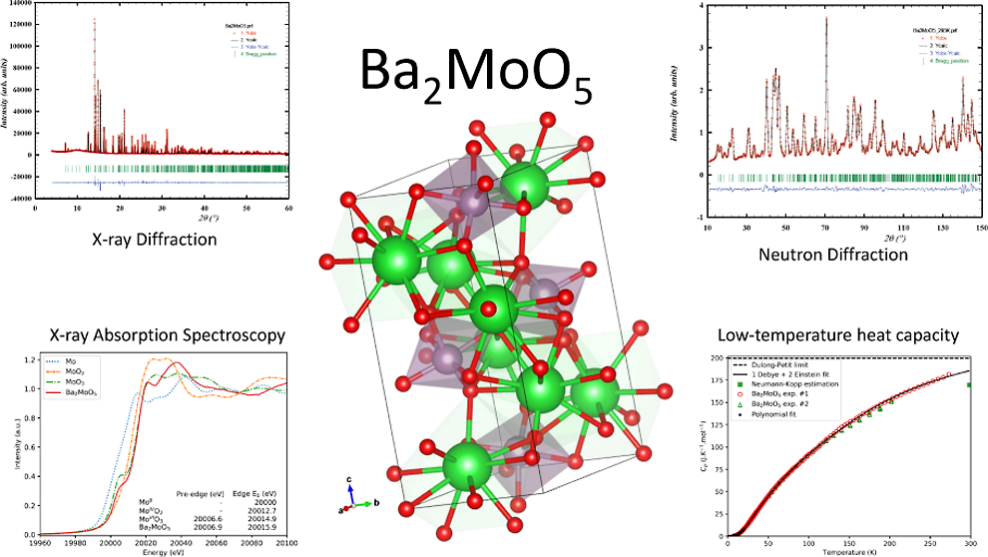

The detailed crystal structure as well as the heat capacity at low temperature and standard entropy of Ba_2_MoO_5_ are reported for the first time. High-resolution X-ray and neutron diffraction were employed to reveal the structural features of this compound. Ba_2_MoO_5_ has a six-coordinated Mo and a strongly negative excess volume with respect to the binary oxides. X-ray absorption near edge structure (XANES) spectroscopy at the Mo K-edge shows Mo to be in the oxidation state 6+. The pre-edge peak in the XANES spectrum indicates a distorted octahedral environment, in line with the results from diffraction studies and FDMNES calculations. The standard entropy and heat capacity of Ba_2_MoO_5_ at 298.15 K, determined with a thermal-relaxation technique, are calculated to be respectively 223.2 ± 7 and 184.7 ± 5 J·K^–1^·mol^–1^. The obtained thermodynamic properties are discussed in the context of the literature reports on molybdate compounds.

## Abstract 2

Ba_2_MoO_5_ was synthesized and its structure was elucidated using synchrotron X-ray and neutron diffraction. The off-center Mo atom causes a pre-edge in the Mo K-edge XANES spectrum. Low-temperature heat capacity and standard entropy were experimentally determined. The structure was found to have a strongly negative excess volume.

## Introduction

Molybdates are a group of compounds with interesting properties in the energy field, for applications in and relevance for catalysis,^1,2^ batteries^3^ and nuclear safety,^4^ because Mo is a high-yield fission product. Besides, molybdates got attention in the field of luminescence as phosphor host materials.^5,6^ Molybdates cannot be considered a uniform group because the structural differences in the molybdate oxyanion are substantial as both tetrahedral and octahedral coordination have been reported in literature. In addition, the arrangement of the polyhedra can range from isolated to corner-, edge- or face-sharing. These different coordination environments will strongly affect the characteristics and properties of the compounds. A striking example are the M_2_MoO_5_ compounds that we encountered in our studies of the MO-MoO_3_ phase diagram (M = Ba,Pb).^4,7^ Pb_2_MoO_5_ forms a framework of Pb–O9 and Mo–O4 polyhedra, whereas Ba_2_MoO_5_ is stated to be orthorhombic with octahedral Mo coordination and isostructural with K_2_VO_2_F_3_ by ref (8) based on private communication with Negas and Roth. The compound Ba_2_MoO_5_ has been mentioned in the literature several times in the 1970s.^8−10^ However, no detailed structural investigation of the atomic positions has been reported, except for a recent reinterpretation of the data reported in ref (8) by Zavodyannyi.^11^

The atomic structures of two tungstate compounds with divalent cations Sr_2_WO_5_ and Ba_2_WO_5_ that are seemingly isostructural with our title compound Ba_2_MoO_5_ were recently found to differ. In comparison to earlier studies, a more physical description of the atomic displacement parameters of Sr_2_WO_5_ was obtained in space group *Pna*2_1_ instead of *Pnma*. The Sr_2_WO_5_ structure exhibits corner-sharing distorted WO_6_ octahedra that form infinite tilted zigzag chains, while Ba_2_WO_5_ has no tilt in the zigzag chain of octahedra.^12^ This raises the question of how the molybdate analog Ba_2_MoO_5_ crystallizes exactly.

In this article, we investigate Ba_2_MoO_5_ using X-ray and neutron-based techniques to solve the open questions on its crystal structure. Moreover, the heat capacity of the compound has been measured at low temperatures, yielding the heat capacity and standard entropy at 298.15 K for the first time.

## Experimental Section

### Synthesis

To synthesize Ba_2_MoO_5_, BaCO_3_ (99%, Fluka) and MoO_3_ (99.5%, Alfa Aesar) were thoroughly mixed in a 2:1 molar ratio. The mixture was heated 3 times in an alumina crucible under Ar atmosphere at 1173 K for a total time of about 70 h with intermittent regrinding. Since the compound attracts moisture from the air in minutes, it was handled and stored in an Ar-filled dry glovebox with H_2_O and O_2_ contents maintained below 5 ppm.

### X-ray and Neutron Diffraction

High resolution synchrotron XRD (sXRD) measurements were collected using the XRD-1 station at the ROBL beamline (BM20) at ESRF.^13,14^ This station is equipped with a 6-circle diffractometer and a Eiger CdTe 500k detector (Dectris). The wavelength of the synchrotron radiation was set to λ = 0.774901 Å. The beam size was 300 × 300 μ m^2^. The sample holder used in the measurements was a 300 μm diameter glass capillary closed with Epoxy glue itself enclosed inside a Kapton tube. Data were collected in transmission mode at 296 K and reduced using the PyFAI software suite.^15^

Neutron diffraction (ND) on Ba_2_MoO_5_ was performed at the PEARL beamline at the Hoger Onderwijs Reactor (HOR) at TU Delft.^16^ The sample was loaded in a vanadium Null-alloy container hermetically closed with a rubber O-ring. A fixed wavelength of 1.667 Å in the angle range 11 ≤ 2θ ≤ 159° was used. Data were collected at 293 K.

The diffraction patterns were analyzed using the Rietveld profile refinement method^17,18^ in the FullProf suite.^19^ Structural visualization was done using the VESTA software.^20^

### X-ray Absorption Near Edge Spectroscopy

X-ray absorption near edge spectroscopy at the Mo K-edge (20 keV) was performed at the INE Beamline^21^ of the KIT Light Source (Karlsruhe, Germany), which has an energy of 2.5 GeV and a maximum current of 170 mA as operating conditions in the KARA storage ring. A Ge(422) double-crystal monochromator was used and the beam spot size of 500 μm by 500 μm was obtained using Rh-coated mirrors. The Ba_2_MoO_5_ sample, as well as MoO_2_ and MoO_3_ references were diluted in BN powder and pressed into pellets for the measurements, enclosed inside a Kapton foil.

XANES spectra were collected at room temperature in fluorescence mode, using a combination of two silicon drift detectors, viz. a Vortex-ME4 (4 elements) and a Vortex-60EX (1 element, Hitachi/SIINT). A step size of 0.75 eV was used in the edge region. The energy *E*_0_ of the edge absorption threshold position was taken at the inflection point of the spectrum. The position of the prepeak was selected from the recorded maximum before the edge. Several acquisitions were performed on the same sample and summed up to improve the signal-to-noise ratio. Before averaging the scans, each spectrum was aligned using the XANES spectrum of a metallic molybdenum reference foil measured in transmission between the second and third ionization chambers. The ATHENA^22^ software was used to analyze the data. The inflection point is taken as the absorption edge position, while the prepeak (when present) was characterized using its peak maximum.

XANES spectra of Ba_2_MoO_5_, BaMoO_4_ and BaMoO_3_ have been calculated with the FDMNES code^23,24^ using the sXRD based Ba_2_MoO_5_ structure presented in this work and previously published structures for BaMoO_4_ and BaMoO_3_.^4^ The calculations were performed with a cluster radius of 7 Å using self-consistent field (SCF) and the PBE96 exchange–correlation potential. The calculated spectra were normalized and the maxima of the whitelines (WL) were aligned to the experimental spectrum of Ba_2_MoO_5_ for better comparability.

### Low-Temperature Heat Capacity

The low-temperature heat capacity of Ba_2_MoO_5_ was measured on pressed pellets of 3 mm diameter with a Quantum Design PPMS 9T machine at JRC Karlsruhe. The heat capacity of the sample equals the difference between the total measured heat capacity and the addenda curve. A pellet of 11.50(10) mg (exp. #1) was encapsulated in 0.824(5) mg Stycast to prevent moisture uptake and improve the thermal heat transfer to the sample platform.^25^ The Stycast contribution as well as the addenda curve were subtracted from the measured heat capacity to obtain the heat capacity of the Ba_2_MoO_5_ material itself. The temperature domain is 7.1–277.2 K.

For the data analysis, two fits were used. Toward the lower limit (*T* < 20 K), the lattice contribution to the heat capacity was fitted to a polynomial expression
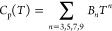
1where *B*_n_ is a constant and *T* is the temperature in K. A linear combination of *n*_D_ times a Debye function (*D*(Θ_D_, *T*)) and *n*_*E*1_ + *n*_*E*2_ times an Einstein function (*E*(Θ_*Ei*_,*T*)) was used to fit the data at higher temperatures (*T* > 20 K). The formula used for fitting in this work reads thus
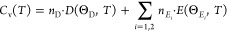
2where the formulas for the Debye (with *x* = Θ_D_/*T*) and Einstein functions are
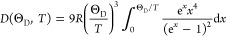
3and
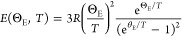
4

The classical Dulong–Petit limit (*C*_v_ = 3*nR*) is hidden in the sum of *n*_D_ + *n*_*E*1_ + *n*_*E*2_, while *C*_p_ ≈ *C*_v_ in the fitted temperature range. The standard entropy at 298.15 K was then calculated by integration of the fits in the temperature domain 0–298.15 K. The total uncertainty in the measured heat capacity is estimated to be at maximum 3%. The uncertainty on the heat capacity was used in the uncertainty determination on the standard entropy.

For verification, the heat capacity of a pellet of Ba_2_MoO_5_ encapsulated in Stycast was also measured on a Quantum Design Versalab machine at TU Delft in the temperature window 50–202 K (exp. #2). The mass of this pellet is 10.40(10) mg with 0.930(10) mg Stycast.

## Results and Discussion

### X-ray and Neutron Diffraction

**Figure 1:**
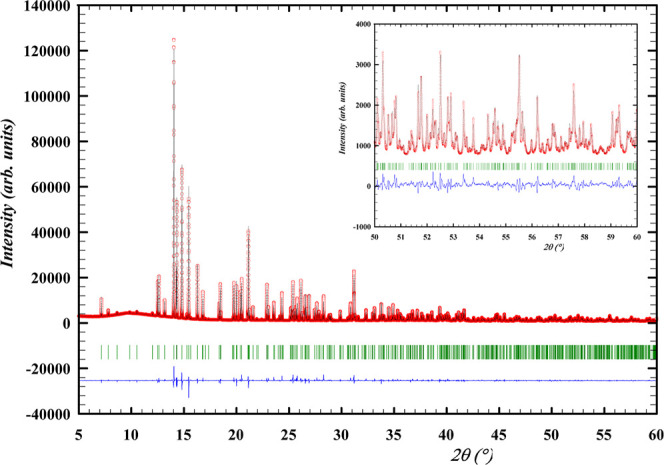
Experimental (*Y*_obs_, in red) and calculated (*Y*_calc_, in black) sXRD patterns of Ba_2_MoO_5_ at ambient temperature. The difference between calculated and experimental intensities *Y*_obs_ – *Y*_calc_ is shown in blue. The angular positions of Bragg reflections are shown in green. Measurement at λ = 0.774901 Å.

**Figure 2:**
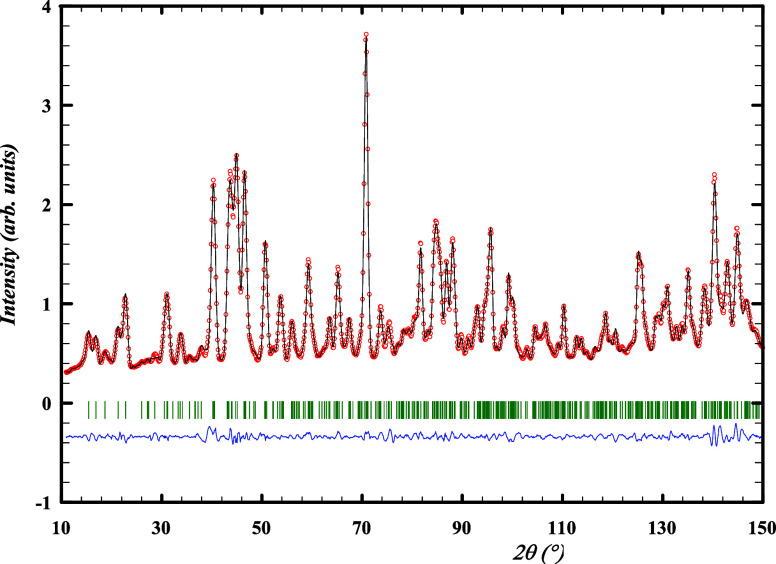
Experimental (*Y*_obs_, in red) and calculated (*Y*_calc_, in black) ND patterns of Ba_2_MoO_5_ at ambient temperature. The difference between calculated and experimental intensities *Y*_obs_ – *Y*_calc_ is shown in blue. The angular positions of Bragg reflections are shown in green. Measurement at λ = 1.667 Å.

A yellow powder was obtained from the above-mentioned synthesis. As a starting point for profile refinement, the atomic positions of K_2_VO_2_F_3_ were used, as has been suggested by Ryan et al.^8,10^ The refinement of the synchrotron X-ray and neutron diffraction data in space group *Pnma* (62) were successful, as can be seen in Figures 1 and 2. In the synchrotron X-ray diffraction pattern, a few minor unexplained peaks (*I*/*I*_max_ < 0.4%) were found at 2θ = 11.4, 12.4, 13.2 and 22.4°. No clear attribution to these peaks can be given, but these may be reaction products of moisture attraction.

The cell parameters as obtained from the refinements are given in Table 1 and compared with values from literature that are obtained by XRD. The cell parameters are in line with literature and each other, though not always within the reported errors. The atomic positions refined from neutron and synchrotron X-ray diffraction are given in Tables 2 and 3, respectively. The isotropic atomic displacement parameters are optimized as well. The values are given in Table 2 for the synchrotron X-ray diffraction data and in Table 3 for the neutron diffraction data.

**Table 1:** Refined Cell Parameters of Ba_2_MoO_5_ Based on sXRD and ND in Space Group *Pnma* (62) (α = β = γ = 90°) in This Work Compared with Literature. sXRD and ND are Synchrotron X-ray Diffraction and Neutron Diffraction, Respectively

method	*a* (Å)	*b* (Å)	*c* (Å)	*V* (Å^3^)
sXRD (this work)	7.41082(10)	5.76170(10)	11.38820(10)	486.26
ND (this work)	7.4009(18)	5.7534(14)	11.3682(19)	484.06
XRD^8^	7.4097(7)	5.7603(6)	11.3096(8)	482.72
XRD^9^	7.412(1)	5.769(1)	11.380(2)	486.61

**Table 2:** Refined Atomic Positions of Ba_2_MoO_5_ and Isotropic Atomic Displacement Parameters Based on Synchrotron X-ray Diffraction^a^

site	Wyckoff	*x*/*a*	*y*/*b*	*z*/*c*	*B* (Å^2^)
Ba1	4c	0.47913(4)	0.25	0.71144(3)	0.371(7)
Ba2	4c	0.18429(5)	0.25	0.41632(3)	0.42(8)
Mo	4c	0.18383(8)	0.25	0.06868(4)	0.228(10)
O1	4c	0.2790(5)	0.25	0.9082(4)	1.18(10)
O2	8d	0.3202(4)	0.0078(4)	0.1186(19)	0.36(5)
O3	4c	0.0211(5)	0.25	0.1906(3)	2.00(10)
O4	4a	0	0	0	0.39(8)

a *R*_p_ = 13.4, *R*_wp_ = 13.8, *R*_exp_ = 4.83, χ^2^ = 8.22.

**Table 3:** Refined Atomic Positions and Isotropic Atomic Displacement Parameters of Ba_2_MoO_5_ Based on Neutron Diffraction^a^

site	Wyckoff	*x*/*a*	*y*/*b*	*z*/*c*	*B* (Å^2^)
Ba1	4c	0.477(4)	0.25	0.71255(3)	0.64(6)
Ba2	4c	0.1865(4)	0.25	0.41604(3)	0.64(5)
Mo	4c	0.1839(3)	0.25	0.07172(2)	0.39(3)
O1	4c	0.2816(4)	0.25	0.91176(3)	0.89(5)
O2	8d	0.3244(2)	0.0086(4)	0.11755(14)	1.21(3)
O3	4c	0.0254(4)	0.25	0.1952(3)	1.28(6)
O4	4a	0	0	0	0.94(4)

a *R*_p_ = 5.7, *R*_wp_ = 6.43, *R*_exp_ = 1.38, χ^2^ = 21.6.

It is interesting to note that the structure of Ba_2_MoO_5_ is closely related to arcanite (K_2_SO_4_), which is adopted by Cs_2_MoO_4_ while a related monoclinic structure is adopted by K_2_MoO_4_.^27^ The “additional” oxygen in the structure promotes an octahedral coordination of Mo compared to the tetrahedral coordination in Cs_2_MoO_4_, without affecting the unit cell volume as the unit cell volumes of Cs_2_MoO_4_, K_2_MoO_4_ and Ba_2_MoO_5_ follow a linear trend as a function of the ionic radius of the (earth-)alkaline cation.

The refined distances in the various polyhedra are summarized in Table 4 and compared to the sum of the Shannon radii of the ions.^26^ The effective ionic radii as reported by Shannon and used here are Ba^2+^(X) = 1.52 Å and Mo^6+^(VI) = 0.59 Å. The oxygen coordination is V (O1, O2 and O3) and VI (O4), the Shannon radii being 1.38 and 1.40 Å for IV- and VI-coordination, respectively. The two Ba-polyhedra are coordinated to 10 O anions and have six different Ba–O bond lengths, ranging from 2.607(4) Å to 2.8906(3) Å (sXRD) or 2.660(5) Å to 2.892(4) Å (ND) for Ba1–O, which is short in comparison with the sum of ionic radii, and from 2.741(3) Å to 3.064(3) Å (sXRD) or 2.733(4) Å to 3.042(4) Å (ND) for Ba2–O, which is on average close to the sum of Shannon ionic radii.

**Table 4:** Bond Lengths for Ba_2_MoO_5_ Obtained From Synchrotron X-ray and Neutron Diffraction Data via Rietveld Refinement^a^

synchrotron X-ray diffraction				
bond	Av. (Å)	Min (Å)	Max (Å)	S.R. (Å)
Ba1–O	2.82	2.607(4)	2.8906(3)	2.92
Ba2–O	2.89	2.741(3)	3.064(3)	2.92
Mo–O1	1.959(5)			1.99
Mo–O2(x2)	1.814(3)			1.99
Mo–O3	1.839(4)			1.99
Mo–O4(x2)	2.1313(5)			1.99

neutron diffraction				
bond	Av. (Å)	Min (Å)	Max (Å)	S.R. (Å)
Ba1–O	2.83	2.660(5)	2.892(4)	2.92
Ba2–O	2.87	2.733(4)	3.042(4)	2.92
Mo–O1	1.956(2)			1.99
Mo–O2(x2)	1.812(3)			1.99
Mo–O3	1.830(4)			1.99
Mo–O4(x2)	2.1414(17)			1.99

a Effective ionic radii for the column “S.R.” are taken from Shannon.^26^

**Figure 3:**
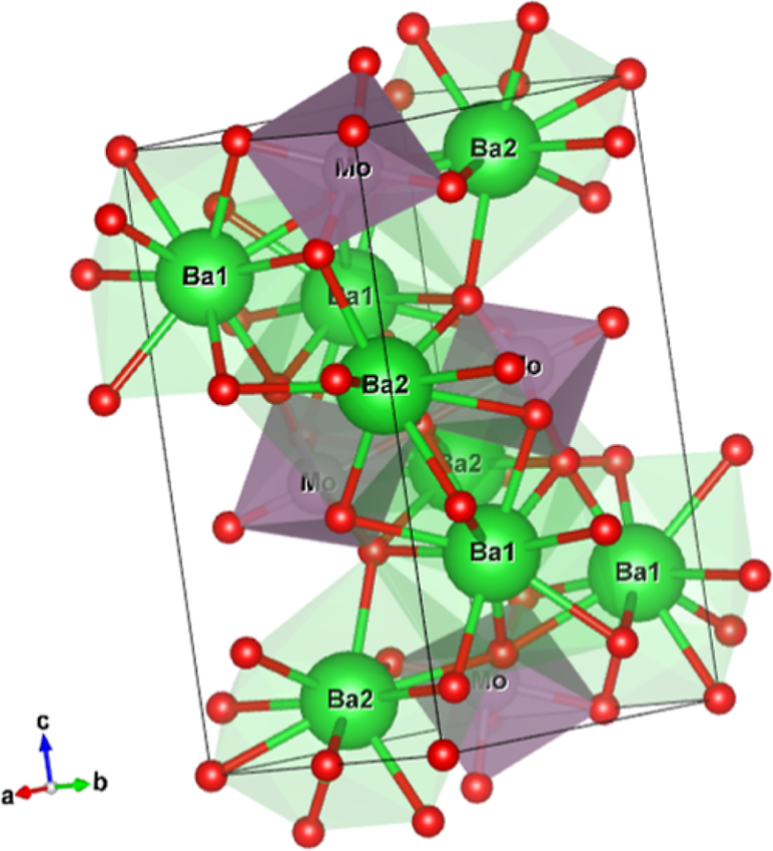
3-Dimensional view of the crystal structure of Ba_2_MoO_5_. The chains of Mo-octahedra can be seen, as well as the off-center Mo-atom. The green, purple and red atoms are Ba, Mo and O, respectively.

**Figure 4:**
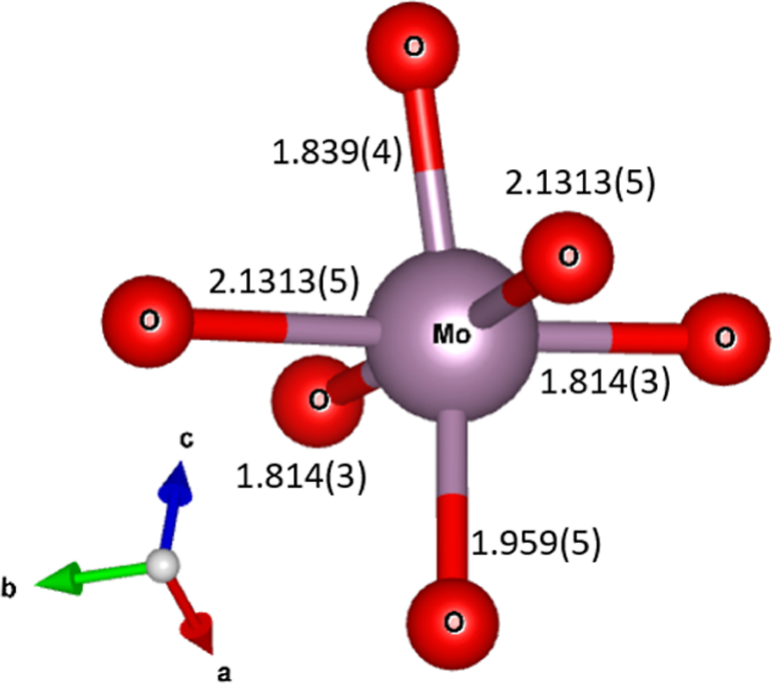
Detailed view of the coordination of Mo showing the off-center Mo-atom and bond lenghts.

**Figure 5:**
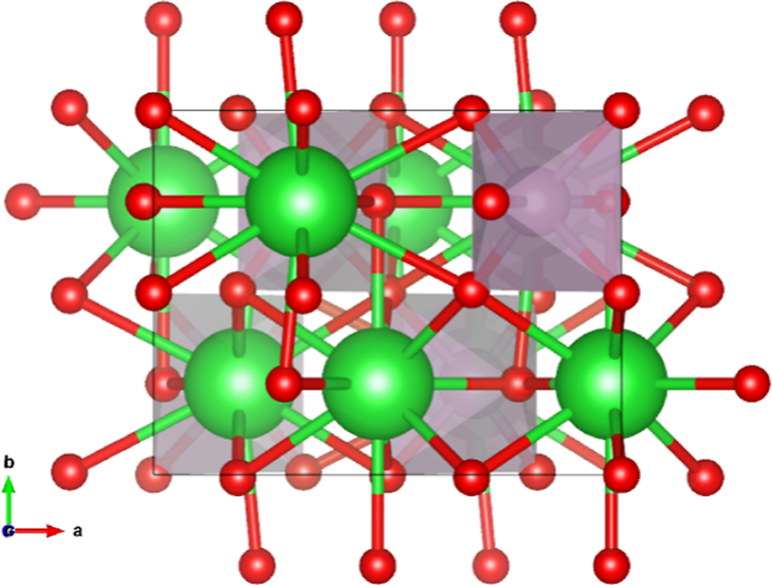
View of the crystal structure of Ba_2_MoO_5_ along the *c*-axis, showing the Mo-octahedra are aligned from this perspective. The green, purple and red atoms are Ba, Mo and O, respectively.

**Figure 6:**
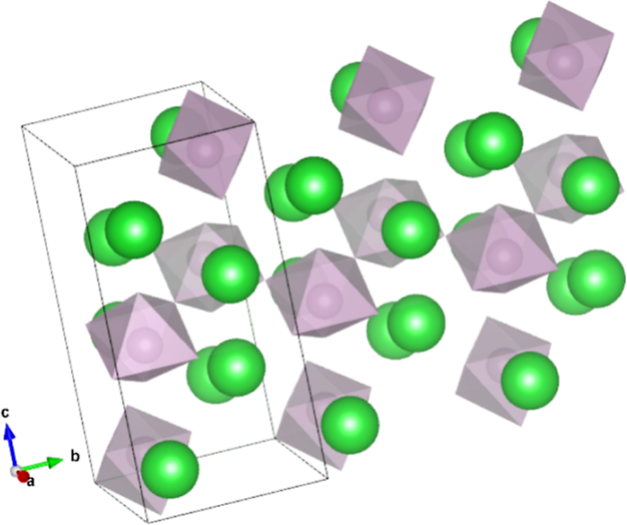
View of the crystal structure of Ba_2_MoO_5_, showing the corner-sharing Mo-octahedra.

The Mo-octahedra have a slightly off-centered Mo-atom, see Figure 3 and the detailed zoom in Figure 4. The Mo-octahedra are corner-sharing via the O4 ions. The Mo–O4–Mo angle equals 180° by symmetry and the chain is thus developing in the *b*-direction in a zigzag mode with nontilted octahedra and with rather long Mo–O4 distances of 2.1313(5) Å (sXRD) or 2.1414(17) Å (ND). The nontilted zigzag alignment can be seen from the top in Figure 5 and the chains are better visible in the three-dimensional perspective in Figure 6. Bond angles are visualized in Figure S.1 and tabulated in Table S.1.

The average Mo–O bond length, 1.9482 Å (sXRD) or 1.9486 Å (ND), is close to but somewhat lower than the sum of Shannon ionic radii (1.97–1.99 Å depending on coordination). Furthermore, it is larger than the Mo–O bond length in undistorted Mo–O6 octahedra (1.92 Å) as derived by Shannon, and thus indicates a distortion.^26^ Instead of using only the tabulated values for Mo^6+^(VI) and O^2–^, the equation by Shannon^26^ for the relation between distortion of the octahedron Δ*a*nd the average Mo^6+^–O bond length *R̅* using the mean-square relative deviation from the average^28^ reads
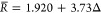
5with^28^
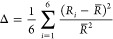
6

Calculation of *R̅* using the distortion parameter Δ based on the bond lengths *R*_*i*_ obtained in this work using sXRD and ND yields *R̅* = 1.94 Å in both cases, showing that the structure of Ba_2_MoO_5_ fits very well the general trend.

The molar volume derived from the crystallographic density of Ba_2_MoO_5_ is lower than the stoichiometric sum of the molar volumes of the constituting oxides. This is different from other known molybdate compounds, as is shown in Table S.2. CoMoO_4_, FeMoO_4_ and NiMoO_4_, in which Mo is octahedrally coordinated like in Ba_2_MoO_5_, have a strongly positive excess volume (>5%) due to the open arrangement of the MoO_6_ and AO_6_ octahedra with a large fraction of corner sharing. The excess volume of the tetrahedrally coordinated arcanite-type alkali molybdates, to which the structure of Ba_2_MoO_5_ is related, is strongly positive (>5%), resulting from a loose arrangement of edge and corner sharing polyhedra. The excess volume of AMoO_4_ molybdate scheelite compounds (A = Ca, Sr, Ba, Pb) varies from slightly negative (−1%) to strongly positive (>5%), in an open arrangement with corner-sharing MoO_4_ tetrahedra. In Pb_2_MoO_5_, the corner-sharing of the polyhedra results in a very open structural arrangement as well. This means that Ba_2_MoO_5_ (and the isostructural Ba_2_WO_5_) is an exceptional case regarding its strongly negative excess volume, resulting from the very effective spatial ordering/packing of the polyhedra in the structure through face sharing made possible by the octahedral coordination of Mo, as can also be seen in Figure S.2.

Recently, Jantz et al.^12^ showed that the compounds Sr_2_WO_5_ and Ba_2_WO_5_ have a different ordering of Mo-octahedra. Sr_2_WO_5_, previously also refined in space group *Pnma* (62), can be more adequately refined in space group *Pna*2_1_ (33), while the earlier assignment of Ba_2_WO_5_ in space group *Pnma* was shown to be accurate. On the atomic level, however, this new description of Sr_2_WO_5_ yielded another view on the atomic displacement parameters. The connection between the W-octahedra is different: for Sr_2_WO_5_ infinite tilted chains were found, while Ba_2_WO_5_ has nontilted chains of octahedra. Herein, an attempt to refine the diffraction data in space group *Pna*2_1_ did not yield an improved refinement when judged from statistical significance; the Mo-octahedra form chains in both space groups. From a principal point of view, no distinction can be made between the space groups *Pnma* (62) and *Pna*2_1_ (33) or, after exchanging the lattice parameters for consistency, *Pn*2_1_*a*. There are several reasons to prefer space group *Pnma*, however: the consistency with existing literature on Ba_2_MoO_5_,^8−11^ the similarity to Ba_2_WO_5_,^12^ the fact that the solution in *Pnma* has less parameters to adjust and only positive atomic displacement parameters (see Tables S.3 and S.4). Thus, Ba_2_MoO_5_ and Ba_2_WO_5_ are structurally similar and both have a strongly negative excess volume.

### X-ray Absorption Near Edge Spectroscopy

**Figure 7:**
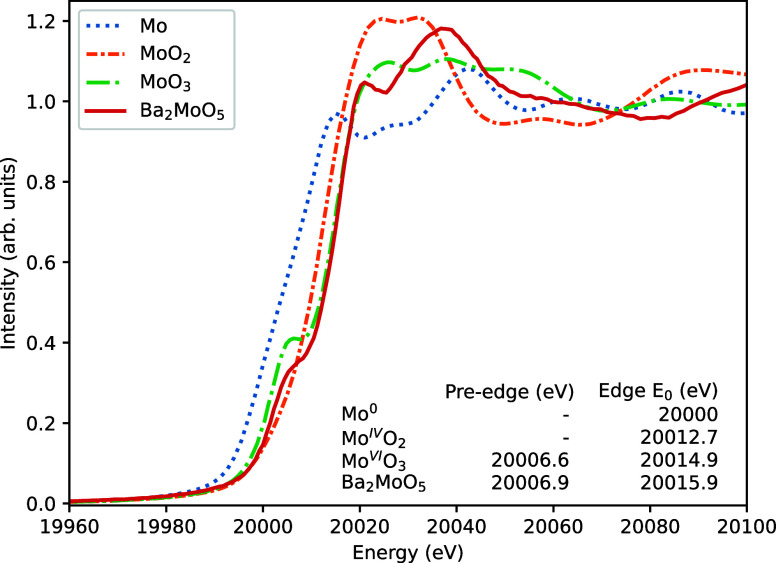
X-ray absorption near edge structure spectra of Ba_2_MoO_5_ measured at the Mo K-edge with reference materials Mo^0^-metal, Mo^IV^O_2_ and α-Mo^VI^O_3_. The uncertainty on the determined edges is ±1 eV.

The oxidation state of Mo in Ba_2_MoO_5_ was determined using X-ray absorption near edge structure spectroscopy. In Figure 7, the collected XANES spectra around the Mo K-edge are shown for Ba_2_MoO_5_ together with the reference materials Mo, MoO_2_ and α-MoO_3_. The derived absorption edge and pre-edge peak features are tabulated in Figure 7 as well. Ba_2_MoO_5_ exhibits an energy shift with respect to Mo-metal and MoO_2_ and the *E*_0_ is very close to that of α-MoO_3_, indicating an oxidation state of 6+.

The pre-edge peak in both 3d and 4d metals can give some insight into the geometry around the atom of which a core electron is excited. The transition moment from Mo(1s) to Mo(4d) is forbidden in the electric dipole approximation, but allowed in the electric quadrupole approximation. However, the intensity of a quadrupole transition is much lower than the intensity of an allowed electrical dipole transition, and the pre-edge peak intensity is to be ascribed to d–p orbital hybridization. Group theoretical considerations show that for O_h_ symmetry there will be no mixing, while for T_d_ symmetry there is. Moreover, distorted octahedral symmetry exhibits a pre-edge peak, but with a lower intensity than compounds with a tetrahedral arrangement.^29^ Herein, for Ba_2_MoO_5_, a pre-edge peak is observed, but less intense than that of α-MoO_3_, which has a distorted octahedral symmetry. In PbMoO_4_,^30^ BaMoO_4_^4^ and Pb_2_MoO_5_,^7^ all having MoO_4_^2–^-tetrahedra, an intense pre-edge peak is visible, while for BaMoO_3_,^4^ which has undistorted octahedral symmetry around Mo, no pre-edge peak is visible. The pre-edge peak indicates clearly a distortion from octahedral symmetry around the Mo-site and the pre-edge peak found with XANES is thus consistent with the Mo-octahedron with off-center Mo as found using diffraction techniques.

**Figure 8:**
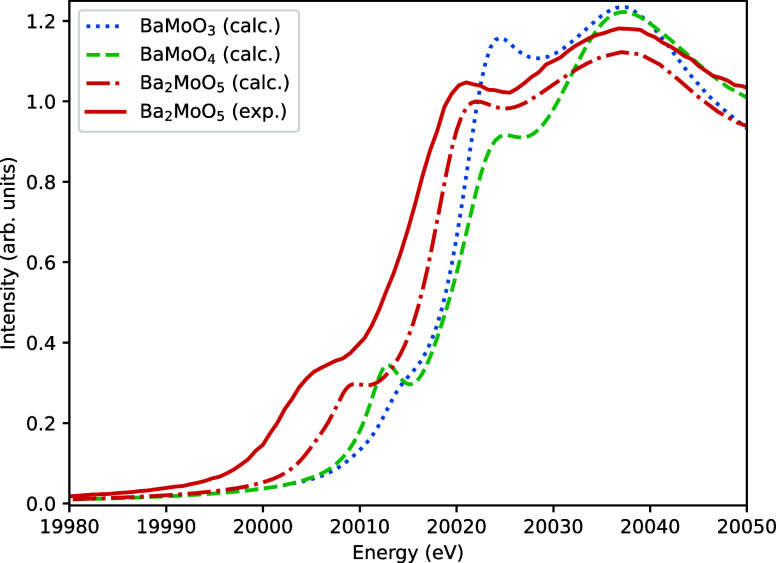
X-ray absorption near edge structure spectra as calculated (calc.) using FDMNES compared to the experimentally measured (exp.) spectrum of Ba_2_MoO_5_. The calculated spectra are aligned to the white line of Ba_2_MoO_5_ (exp.).

The behavior of the pre-edge is moreover confirmed by the calculated XANES spectra of Ba_2_MoO_5_, BaMoO_4_ and BaMoO_3_, which are shown in Figure 8. Here BaMoO_3_ is only showing a small shoulder, BaMoO_4_ has a clear pre-edge peak and Ba_2_MoO_5_ is lying in the middle. Additionally, the calculated Ba_2_MoO_5_ spectrum is in good overall agreement with the experimental data, further validating the structural model of Ba_2_MoO_5_.

### Heat Capacity and Standard Entropy

**Figure 9:**
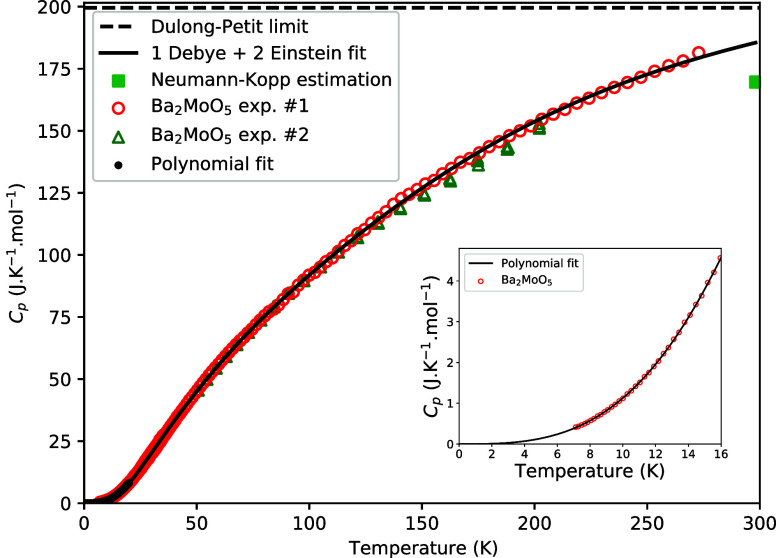
Heat capacity of Ba_2_MoO_5_ as measured in this work and fitted to a polynomial fit and a combined Debye–Einstein fit with comparison to the classical Dulong–Petit limit and Neumann–Kopp estimation. See the main text for the explanation of the fits.

**Figure 10:**
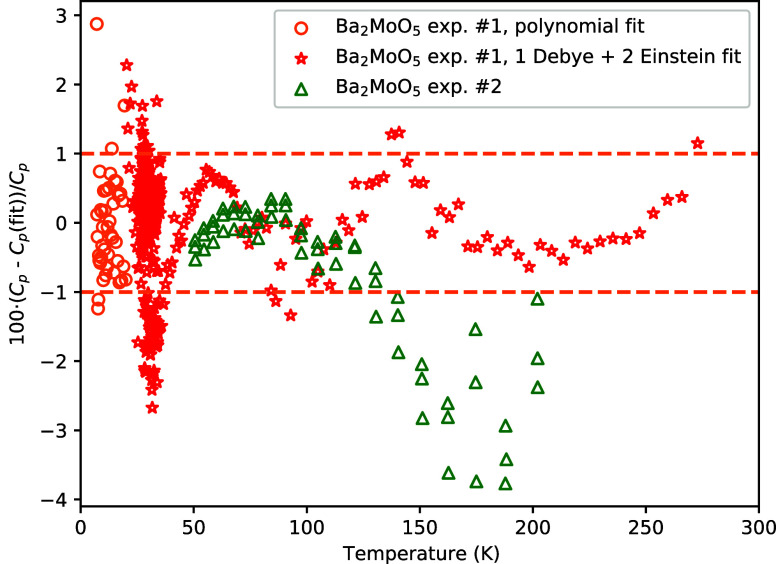
Relative difference between the experimental heat capacity data and the polynomial and combined Debye–Einstein fit. See the main text for the explanation of the fits.

The low temperature heat capacity of Ba_2_MoO_5_ between 7.1 and 277.2 K is shown in Figure 9. No anomalies are observed in the studied temperature window and the results of the two measurements with different instruments are in good agreement. Mathematical fits as explained *supra* were made; the fitting parameters are given in Table 5. The relative difference between the fits and the experimental data is shown in Figure 10. Using the data below 10 K, the heat capacity in a plot of *C*_p_/*T* vs *T*^2^ yields a curve which extrapolates to 0, meaning there is no electronic contribution to the heat capacity and Ba_2_MoO_5_ is an insulating material (see also Figure S.3).

**Table 5:** Fitting Parameters for the Low-Temperature Heat Capacity of Ba_2_MoO_5_

parameter	value
temp. range/K	7.1–20
*B*_3_/mJ·mol^–1^·K^–4^	1.096 × 10^–3^ ± 7 × 10^–5^
*B*_5_/mJ·mol^–1^·K^–6^	4.509 × 10^–7^ ± 9 × 10^–7^
*B*_7_/mJ·mol^–1^·K^–8^	–1.289 × 10^–9^ ± 4 × 10^–9^
*B*_9_/mJ·mol^–1^·K^–10^	–6.253 × 10^–13^ ± 5 × 10^–12^
temp. range/K	20–277.2
*n*_D_/mol	2.71 ± 0.07
*n*_*E*1_/mol	2.53 ± 0.10
*n*_*E*2_/mol	3.88 ± 0.11
Θ_D_/K	169.4 ± 2.1
Θ_*E*1_/K	316.5 ± 11
Θ_*E*2_/K	711.8 ± 17

**Table 6:** Comparison of the Standard Entropy and Heat Capacity at 298.15 K of Ba_2_MoO_5_ as Compared to an Estimation by Glasser^31^ and an Optimization by Smith et al^4^^a^

parameter	value
*S*_*m*_^◦^ (298.15 K) (this work)	223.2 ± 7
*S*_*m*_^◦^ (298.15 K) (Calphad model^4^)	223.7
*S*_*m*_^◦^ (298.15 K) (estimation^31^)	200.0
*C*_p_ (298.15 K) (this work)	184.7 ± 5
*C*_p_ (298.15 K) (Neumann–Kopp)	169.2

a All numbers in units of J·K^–1^ mol^–1^.

**Figure 11:**
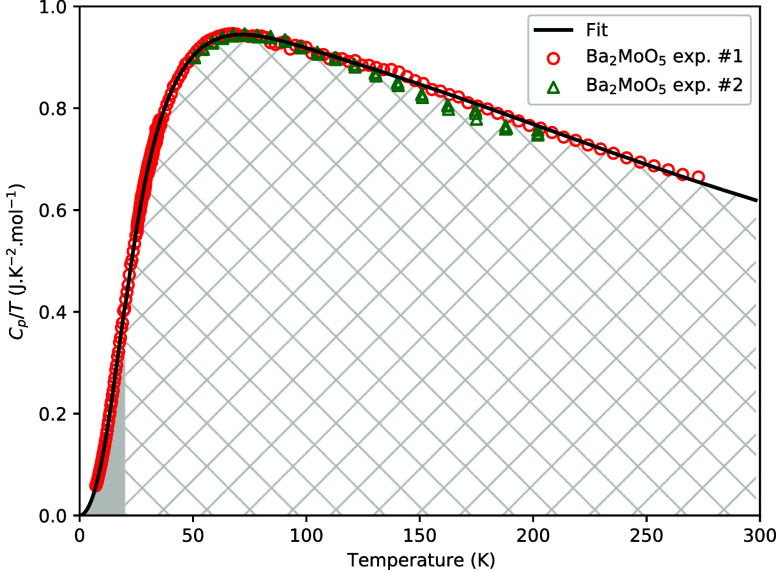
Heat capacity as plotted in *C*_p_/*T* vs *T*. The shaded areas represent the contributions to the standard entropy as calculated using the two fits in their respective domains.

In Figure 11, the shaded areas represent the contribution to the entropy of the polynomial fit and the combined Debye–Einstein fit in the fully shaded and dashed areas, respectively. The obtained standard entropy is *S*_*m*_^◦^ (298.15 K, Ba_2_MoO_5_) = 223.2 ± 7 J·K^–1^·mol^–1^. The heat capacity obtained by extrapolation is *C*_*p*_(298.15 K, Ba_2_MoO_5_) = 184.7 ± 5 J·K^–1^·mol^–1^.

The obtained standard entropy value agrees very well with the value Smith et al.^4^ optimized in a thermodynamic assessment of the Ba–Mo–O system, while the estimation method of Glaser^31^ is quite far off. The heat capacity at 298.15 K is higher than the estimation of Smith et al., which was based on a Neumann–Kopp estimation^4^ (Table 6).

The Neumann–Kopp approximation yields 169.2 J·K^–1^·mol^–1^, based on values for BaO (47.06 ± 0.1 J·K^–1^·mol^–1^)^32^ and α-MoO_3_ (75.07 J·K^–1^·mol^–1^).^33^ The failure of the Neumann–Kopp estimation for the heat capacity at 298.15 K can tentatively be explained by the fact that the heat capacity of layered α-MoO_3_ is not yet approaching its Dulong–Petit limit (C_v_ = 3*nR*) at 298.15 K, unlike BaO and, as it turned out, the title compound Ba_2_MoO_5_. A check for CaMoO_4_, SrMoO_4_, BaMoO_4_, PbMoO_4_, NiMoO_4_, FeMoO_4_ and Pb_2_MoO_5_ showed that the difference to the Neumann–Kopp rule using α-MoO_3_ is exceptionally large, as can be found in Table S.5. Moreover, all cases except Ba_2_MoO_5_ and Pb_2_MoO_5_ have a negative difference to the Neumann–Kopp rule (again, PbO is a layered compound that does not reach the Dulong–Petit limit at room temperature). Although the stoichiometry of Pb_2_MoO_5_ hints at a close relation with Ba_2_MoO_5_, the Mo-coordination in both compounds is different, which results in different lattice vibrations. To the best of our knowledge, no full low-temperature heat capacity of the compounds Ba_2_WO_5_ or Sr_2_WO_5_ or values at ambient temperature are available, making a comparison to these compounds impossible. The comparatively high heat capacity for Ba_2_MoO_5_ asks thus for more investigation, either by measurements of the low-temperature heat capacity of Mo- or W-analogous compounds, or by complementary studies into the phonon behavior of Ba_2_MoO_5_ at room temperature and below using techniques such as Raman spectroscopy or inelastic neutron scattering.

## Conclusions

Ba_2_MoO_5_ has been synthesized and structurally studied using neutron and X-ray diffraction. The compound was found to crystallize in space group *Pnma*. It has slightly distorted Mo-octahedra with off-center Mo that form corner-sharing nontilted zigzag chains. The pre-edge peak in the X-ray absorption spectrum indicates a distorted octahedral environment as well, while the oxidation state as determined using the absorption edge was found to be 6+. FDMNES calculations of the XANES spectrum further validated the structural model of Ba_2_MoO_5_. The standard entropy and heat capacity of Ba_2_MoO_5_ at 298.15 K were successfully determined. The obtained values are compared to available literature data for molybdate compounds and show that Ba_2_MoO_5_ is anomalous in terms of a strongly negative excess volume and strongly positive excess heat capacity, both relative to the binary oxides. It is tempting to attribute this to the peculiar arcanite K_2_MoO_4_-like structure in which the additional oxygen promotes the octahedral coordination of Mo compared to the tetrahedral coordination in arcanite, though achieved through a substantial deformation of the octahedra that includes off-center displacement of the Mo. The resulting face-sharing Ba- and Mo-polyhedrons build a dense structure.

## Supporting Information Available

The data for the low-temperature heat capacity of Ba_2_MoO_5_ are attached as an Excel file (XLSX)
Supporting Information to “Elucidation of the Off-Center Displaced Mo in Octahedral Coordination in Ba_2_MoO_5_” (PDF)
