# Supplementary material for: Elucidation of the Off-Center Displaced Mo in Octahedral Coordination in Ba2MoO5
Source: Inorg Chem. 2025 Jan 1;64(1):674–81. doi: 10.1021/acs.inorgchem.4c03617 (PMC11734122; doi:10.1021/acs.inorgchem.4c03617)
Supplement: Supplementary file 2 — ic4c03617_si_002.pdf [file ic4c03617_si_002.pdf]

Supporting Information to 'Elucidation of the Off-centre Displaced Mo in Octahedral Coordination in Ba_2MoO_5'

Andries van Hattem,[†] Laurent de Geus,[†] Ana Sacristán,[†] Robert Dankelman,[†] Sebastian Couweleers,[†] Christoph Hennig,[‡] Jean-Christophe Griveau,[¶] Eric Colineau,[¶] Kathy Dardenne,[§] Jörg Rothe,[§] Tim Pruessmann,[§] Rudy J.M. Konings,[†] and Anna L. Smith[*,†]

†*Radiation Science & Technology Department, Faculty of Applied Sciences, Delft University of Technology, Mekelweg 15, Delft, 2629JB, The Netherlands*

‡*ESRF, The European Synchrotron, 71 Avenue des Martyrs, Grenoble Cedex 9, CS40220, 38043, France*

¶*European Commission, Joint Research Centre, Karlsruhe, Germany*

§*Karlsruhe Institute of Technology (KIT), Institute for Nuclear Waste Disposal (INE), Radionuclide Speciation Department, Hermann-von-Helmholtz-Platz 1, Eggenstein-Leopoldshafen, 76344, Germany*

E-mail: a.l.smith@tudelft.nl

Bond angles around Mo and in chains

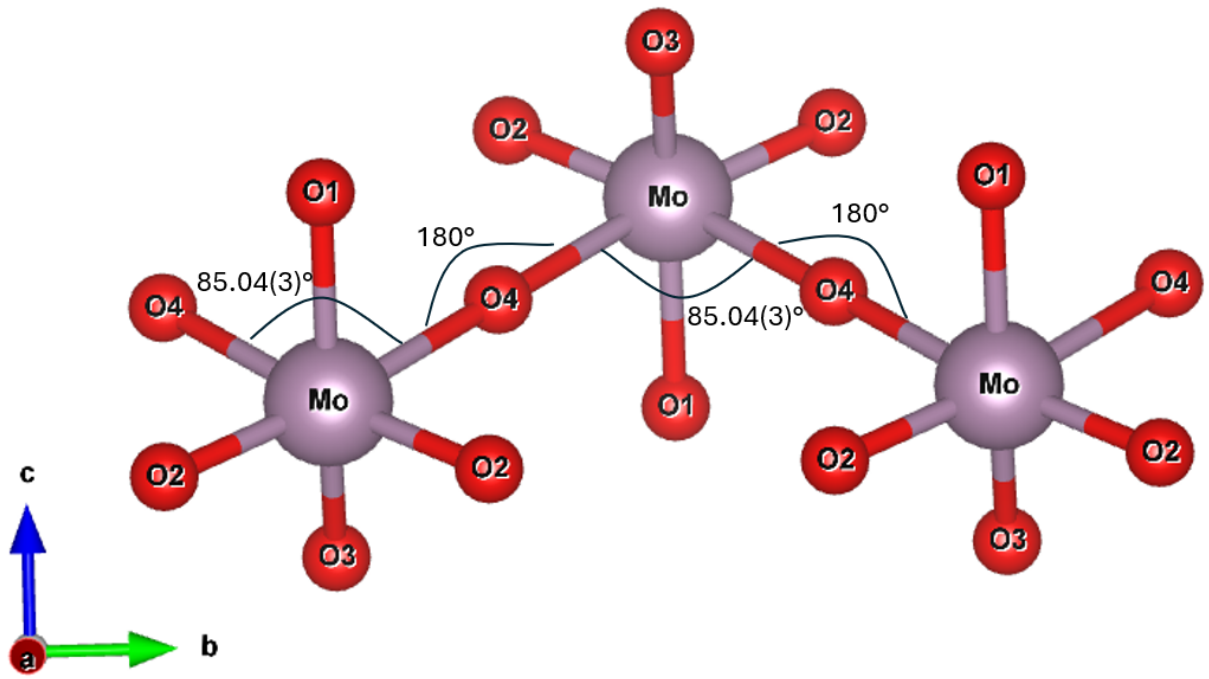

Figure S.1: Visualisation of the bond angles around Mo and in the chains based on synchrotron X-ray diffraction. The connection in the chains is fixed by symmetry via 180°-angle Mo-O4-Mo bonds in space group *Pnma*, which is depicted in Figure S.1. In the discarded space group $Pna2_1$, this bridging oxygen made angles of 172(10)° (sXRD) and 169.0753(8)° (ND).

Table S.1: Adjacent (adj.) and opposite (opp.) neighbor O-Mo-O angles in *Pnma* as measured from synchrotron X-ray diffraction (sXRD) and neutron diffraction (ND) using VESTA.[1]

Connection	Atoms	Angle (°) sXRD	Angle (°) ND	
adj.	O1-Mo-O2	95.27(11)	93.16(12)	2x
adj.	O1-Mo-O4	83.56(9)	83.18(10)	2x
adj.	O2-Mo-O2	100.55(17)	100.11(17)	
adj.	O2-Mo-O4	87.20(9)	87.67(7)	2x
adj.	O4-Mo-O4	85.04(3)	84.39(9)	
opp.	O1-Mo-O3	160.13(17)	161.81(19)	
opp.	O2-Mo-O4	172.24(9)	171.60(12)	2x

Excess Molar Volume

Table S.2: Excess molar volume calculations. The molar volume V_m values are calculated by (molar mass)/(density) using values from CRC Handbook of Chemistry and Physics (97th edition, 2017) unless otherwise stated. Most other values are found via Pearsons Crystal Database.[12] For MoO_3, this yields[13] 143.9 g·mol^{-1} / 4.7 g·cm^{-3} = 30.6 cm^3·mol^{-1}. See Figure S.2 for visualisations. * = This work.

Compound	Mo Coord.	$V_m(A^{II}O)$	V_m (additive)	V_m(experimental)	ΔV_m (%)
Ba_2MoO_5	VI	25.6[2]	84.2	73.3*	-10.5
Ba_2WO_5	VI	25.6[2]	85.8	72.9	-12.6
$CoMoO_4$-type					
$FeMoO_4$	VI	12.0	42.6	46.2[3]	8.5
$NiMoO_4$	VI	11.1	41.7	44.3[4]	6.2
$CoMoO_4$	VI	11.5	42.1	45.4[5]	7.7
Arcanite-related					
K_2MoO_4	IV	40.1[6]	70.7	76.8	8.6
Rb_2MoO_4	IV	46.2[7]	76.8	86.4	12.5
Cs_2MoO_4	IV	59.6[7]	90.2	97.2	7.8
Scheelite-type					
$PbMoO_4$	IV	23.9	54.5	53.9[8]	-1.1
$CaMoO_4$	IV	16.8	47.4	47.0[9]	-1.0
$SrMoO_4$	IV	20.3	50.9	52.7[10]	3.4
$BaMoO_4$	IV	25.6[2]	57.4	59.8	6.3
Pb_2MoO_5	IV	23.9	78.4	81.8[11]	4.3

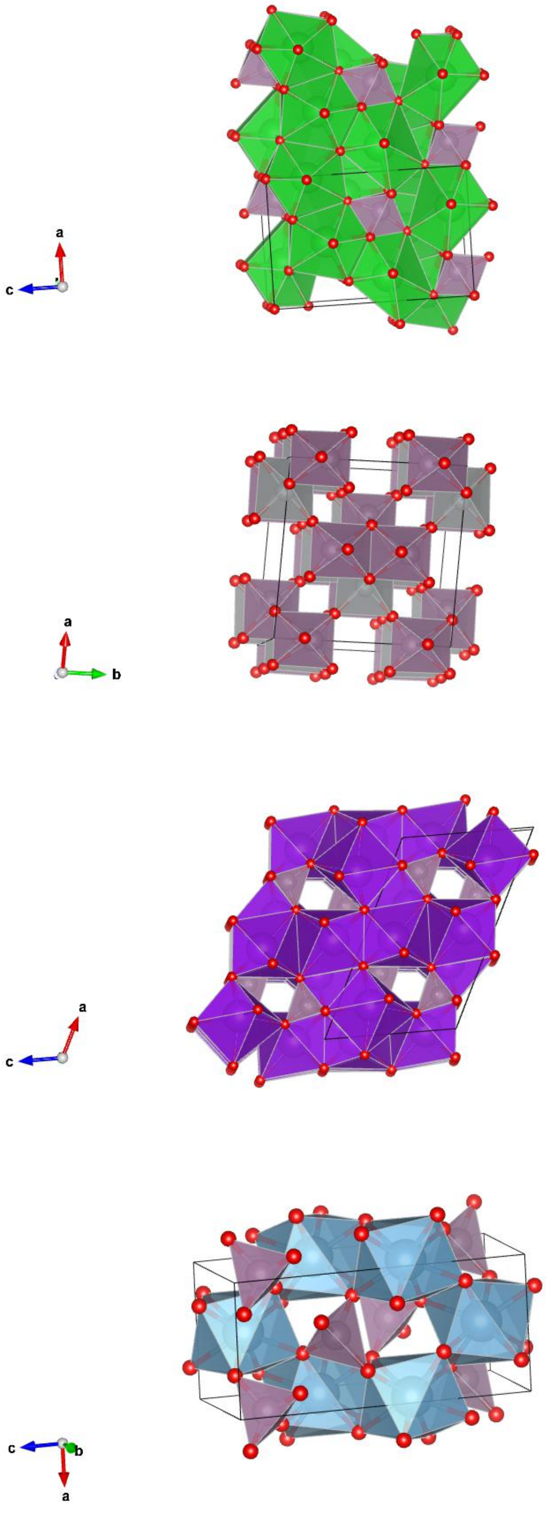

Figure S.2: Example crystal structures to Table S.4. From top to bottom are represented Ba_2MoO_5, $CoMoO_4$-type (shown: $NiMoO_4$-C2/m), arcanite-type (shown: K_2MoO_4-C2/m) and scheelite-type (shown: $CaMoO_4$(powellite)). Pictures from PCD[12] visualised using VESTA.[1]

Isotropic Atomic Displacement Parameters

Table S.3: Refined isotropic atomic displacement parameters of Ba_2MoO_5 for the various refinement attempts, all in Å^2. ND and SD are Neutron Diffraction and Synchrotron Diffraction, respectively.

Pnma (62)				*Pna*2$_1$ (33)			
Site	Wyckoff	*B* (ND)	*B* (SD)	Site	Wyckoff	*B*(ND)	*B*(SD)
Ba1	4c	0.64(6)	0.371(7)	Ba1	4a	0.69(16)	0.527(7)
Ba2	4c	0.64(5)	0.42(8)	Ba2	4a	0.65(19)	0.593(8)
Mo	4c	0.39(3)	0.228(10)	Mo	4a	1.32(16)	0.368(12)
O1	4c	0.89(5)	1.18(10)	O1	4a	1.28(12)	-0.32(11)
O2	8d	1.21(3)	0.36(5)	O2a	4a	1.23(18)	1.12(14)
				O2b	4a	1.6(2)	0.46(13)
O3	4c	1.28(6)	2.00(10)	O3	4a	2.5(3)	-0.62(13)
O4	4a	0.94(4)	0.39(8)	O4	4a	0.52(18)	0.80(18)

Table S.4: Refined isotropic atomic displacement parameters in *Pnma* for of Ba_2MoO_5 compared to Ba_2WO_5.[14] All *B* in Å^2. ND, XRD and SD are Neutron Diffraction, X-ray Diffraction and Synchrotron Diffraction, respectively.

Ba_2MoO_5				Ba_2WO_5		
Site	Wyckoff	*B* (ND)	*B* (SD)	Site	Wyckoff	*B*(XRD)
Ba1	4c	0.64(6)	0.371(7)	Ba1	4c	0.248(3)
Ba2	4c	0.64(5)	0.42(8)	Ba2	4c	0.279(3)
Mo	4c	0.39(3)	0.228(10)	W	4c	0.172(3)
O1	4c	0.89(5)	1.18(10)	O1	4c	0.466(32)
O2	8d	1.21(3)	0.36(5)	O2	8d	0.411(32)
O3	4c	1.28(6)	2.00(10)	O3	4c	0.418(32)
O4	4a	0.94(4)	0.39(8)	O4	4a	0.418(24)

Excess Heat Capacity

Table S.5: All C_p(298.15 K) in J·K^{-1}·mol^{-1}. DP = Dulong-Petit limit; NK = Neumann-Kopp approximation. * = This work. The value used for $C_p(MoO_3)$ in the Neumann-Kopp approximation is 75.07 J·K^{-1}·mol^{-1}.[24]

Compound	Mo Coord.	C_p(DP)	$C_p(A^{II}O)$	C_p (NK)	C_p (exp.)	ΔC_p (%)
Ba_2MoO_5	VI	199.5	47.06[15]	169.19	184.7±5*	9.2
Pb_2MoO_5	IV	199.5	45.77[16]	166.61	172±5[8]	3.2
$FeMoO_4$	VI	149.7	48.97[17]	124.04	118.4[18]	-4.5
$NiMoO_4$	VI	149.7	44.40[19]	119.47	109[20]	-8.8
$MgMoO_4$	IV	149.7	37.11[16]	112.18	110.55[21]	-1.5
$CaMoO_4$	IV	149.7	42.12[16]	117.19	114.31±0.8[22]	-2.5
$SrMoO_4$	IV	149.7	45.41[16]	120.48	116.41[21]	-3.4
$BaMoO_4$	IV	149.7	47.06[15]	122.13	118.35[21]	-3.1
					126.4±3.8[23]	3.5
$PbMoO_4$	IV	149.7	45.77[16]	120.84	118.8[8]	-1.6

Extrapolation Heat Capacity to 0 K

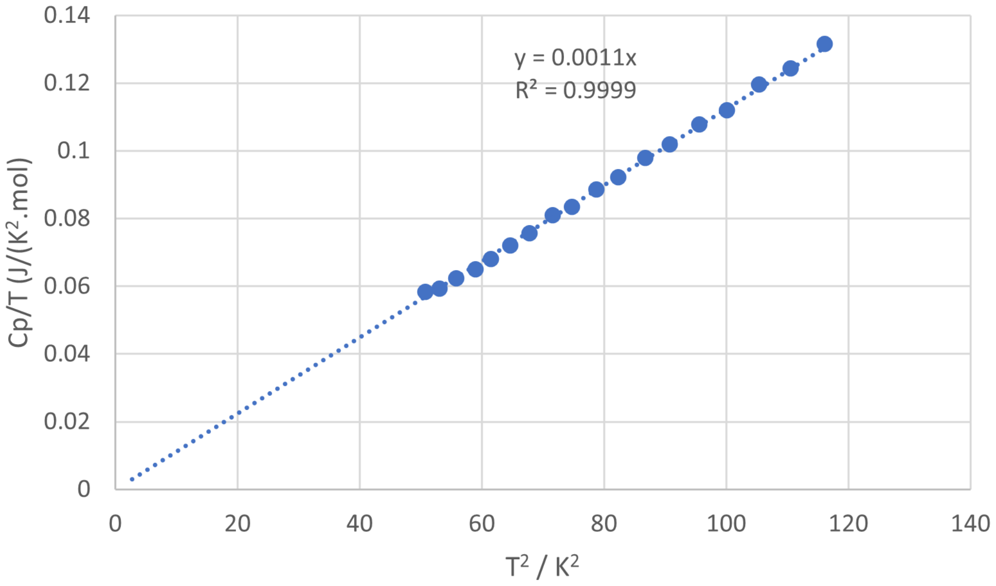

Figure S.3: Extrapolation of the heat capacity to 0 K

References

(1) Momma, K.; Izumi, F. VESTA 3 for three-dimensional visualization of crystal, volumetric and morphology data. *Journal of applied crystallography* **2011**, *44*, 1272–1276.

(2) Taylor, D. Thermal expansion data. I: Binary oxides with the sodium chloride and wurtzite structures, MO. *Transactions and journal of the British Ceramic Society* **1984**, *83*, 5–9.

(3) Sleight, A.; Chamberland, B. t.; Weiher, J. Magnetic, Moessbauer, and structural studies on three modifications of $FeMoO_4$. *Inorganic Chemistry* **1968**, *7*, 1093–1098.

(4) Smith, G. The crystal structures of cobalt molybdate $CoMoO_4$ and nickel molybdate $NiMoO_4$. *Acta Crystallographica* **1962**, *15*, 1054–1057.

(5) Sleight, A.; Chamberland, B. Transition metal molybdates of the type $AMoO_4$. *Inorganic Chemistry* **1968**, *7*, 1672–1675.

(6) Zintl, E.; Harder, A.; Dauth, B. Gitterstruktur der oxyde, sulfide, selenide und telluride des lithiums, natriums und kaliums. *Zeitschrift für Elektrochemie und angewandte physikalische Chemie* **1934**, *40*, 588–593.

(7) Helms, A.; Klemm, W. Die Kristallstrukturen von Rubidium-und Cäsium-Oxyd. *Zeitschrift für anorganische und allgemeine Chemie* **1939**, *242*, 33–40.

(8) van Hattem, A.; Dankelman, R.; Colineau, E.; Griveau, J.-C.; Dardenne, K.; Rothe, J.; Couweleers, S.; Konings, R. J.; Smith, A. L. Experimental investigations and thermodynamic modelling of the ternary system Pb-Mo-O. *Journal of Alloys and Compounds* **2024**, 175588.

(9) Aleksandrov, V.; Gorbatyii, L.; Ilyukhin, V. Crystal structure of powellite $CaMoO_4$. *Soviet Physics-Crystallography* **1968**, *13*, 512–13.

(10) Deshpande, V.; Suryanarayana, S. Thermal expansion of strontium molybdate. *Acta Crystallographica Section A: Crystal Physics, Diffraction, Theoretical and General Crystallography* **1972**, *28*, 94–95.

(11) Mentzen, B.; Latrach, A.; Bouix, J.; Hewat, A. The crystal structures of $PbO \cdot PbXO_4$ (X= S, Cr, Mo) at 5K by neutron powder profile refinement. *Materials research bulletin* **1984**, *19*, 549–554.

(12) Villars, P.; Cenzual, K.; others Pearson's crystal data: crystal structure database for inorganic compounds. *(No Title)* **2007**,

(13) Atuchin, V.; Gavrilova, T.; Grigorieva, T.; Kuratieva, N.; Okotrub, K.; Pervukhina, N.; Surovtsev, N. Sublimation growth and vibrational microspectrometry of α-MoO3 single crystals. *Journal of crystal growth* **2011**, *318*, 987–990.

(14) Jantz, S. G.; Pielnhofer, F.; Dialer, M.; Höppe, H. A. On Tungstates of Divalent Cations (I)–Structural Investigation and Spectroscopic Properties of $Sr_2[WO_5]$ and $Ba_2[WO_5]$. *Zeitschrift für anorganische und allgemeine Chemie* **2017**, *643*, 2024–2030.

(15) Cordfunke, E. H. P.; van der Laan, R.; van Miltenburg, J. Thermophysical and thermochemical properties of BaO and SrO from 5 to 1000 K. *Journal of Physics and Chemistry of Solids* **1994**, *55*, 77–84.

(16) Chase, M. W.; National Information Standards Organization (US) *NIST-JANAF thermochemical tables*; American Chemical Society Washington, DC, 1998; Vol. 9.

(17) Lemire, R.; Berner, U.; Musikas, C.; Palmer, D.; Taylor, P.; Tochiyama, O. Chemical thermodynamics of iron, Part 2. **2013**,

(18) Lyon, W. G.; Westrum Jr, E. F. Heat capacities of alpha-iron (II) molybdate and iron (II) molybdate-II from 5 to 350 K. *The Journal of Chemical Thermodynamics* **1975**, *7*, 741–757.

(19) Gamsjäger, H.; Bugajski, J.; Preis, W.; others *Chemical thermodynamics of nickel*; Elsevier Amsterdam, 2005.

(20) Morishita, M.; Navrotsky, A. Calorimetric study of nickel molybdate: heat capacity, enthalpy, and Gibbs energy of formation. *Journal of the American Ceramic Society* **2003**, *86*, 1927–1932.

(21) Morishita, M.; Kinoshita, Y.; Nozaki, A.; Yamamoto, H. Thermodynamic properties for $MMoO_4$ (M= Mg, Sr and Ba) as the end-members of the yellow phases formed in the nuclear fuel waste glasses. *Applied Geochemistry* **2018**, *98*, 310–320.

(22) Morishita, M.; Kinoshita, Y.; Houshiyama, H.; Nozaki, A.; Yamamoto, H. Thermodynamic properties for calcium molybdate, molybdenum tri-oxide and aqueous molybdate ion. *The Journal of Chemical Thermodynamics* **2017**, *114*, 30–43.

(23) Smith, A.; Rutten, M.; Herrmann, L.; Epifano, E.; Konings, R.; Colineau, E.; Griveau, J.-C.; Guéneau, C.; Dupin, N. Experimental studies and thermodynamic assessment of the Ba-Mo-O system by the CALPHAD method. *Journal of the European Ceramic Society* **2021**, *41*, 3664–3686.

(24) Gurvich, L.; Polyshchuk, V.; Yorish, V.; Yungman, V. The IVTAN Data Bank on the Thermodynamic Properties of Individual Substances. 1981.
